# The integration of mental health and substance abuse SBIRT among SAMHSA grantees

**DOI:** 10.1186/1940-0640-7-S1-A5

**Published:** 2012-10-09

**Authors:** Manu Singh, Jennifer Kasten, Susan Hayashi, Raphael Gaeta, Rossen Tsanov, Erin Schmeider

**Affiliations:** 1 JBS International, Inc., North Bethesda, MD, USA

## 

Since 2004, the Substance Abuse & Mental Health Services Administration (SAMHSA) has funded three cohorts of state grantees to implement screening, brief intervention, and referral to treatment (SBIRT), a public-health model to identify and address risky alcohol or other drug use. A sustainability study of the Cohort-I SAMHSA SBIRT grantees was conducted using telephone interviews, on-site interviews, and structured observations. Results indicated that seven of the nine Cohort-I sites sustained SBIRT services for alcohol and drugs following SAMHSA funding by incorporating services into a broader wellness model that screened for behavioral and mental as well as physical health. Under this holistic model, SBIRT staff of sustained programs reported greater integration into the host medical facility, buy-in from medical staff, and increased potential for third-party reimbursement for SBIRT services. The literature and findings from the World Health Organization further confirm the effectiveness of adapted SBIRT models for mental health in reducing depressive symptoms and reducing anxiety. The findings from the SBIRT sustainability study and the literature warrant further study among current SAMHSA SBIRT grantees to examine the status of mental health in the delivery of SBIRT for substance use. SAMHSA has begun work with grantees to integrate mental-health services as part of the SBIRT process. To examine grantee technical-assistance needs related to this, in June 2011, SAMHSA SBIRT grantees will report on the extent to which they are screening and delivering services for mental health, how services are delivered, and what grantees’ perceptions are of the needs for mental-health services among their populations and the importance of addressing those needs. The results of this report will be presented as they relate to the Cohort-1 sustainability study findings, the current literature, and future directions for SBIRT program sustainability.

